# Clinical Performance of the Brain4care System for Noninvasive Detection of Intracranial Hypertension

**DOI:** 10.1007/s12028-025-02273-6

**Published:** 2025-04-28

**Authors:** Gustavo Frigieri, Thauan Leandro Gonçalves, Gabriela Nagai Ocamoto, Rodrigo de AP Andrade, Bruno Cezar de Padua, Danilo Cardim

**Affiliations:** 1 brain4care Brazil, São Carlos, Brazil; 2 brain4care USA, Johns Creek, GA USA

**Keywords:** Intracranial compliance, Intracranial pressure, Intracranial hypertension, Noninvasive monitoring

## Abstract

**Background:**

Noninvasive methods for detecting intracranial hypertension (IH) are of growing importance in clinical settings. This study evaluates the clinical performance of the brain4care (B4C) System, which captures pulsatile cranial expansions that reveal a surrogate intracranial pressure (ICP) waveform and subsequently derives the P2/P1 ratio and time-to-peak (TTP) parameters to predict IH.

**Methods:**

This was a retrospective study conducted across multiple centers that included a total of 124 patients. Invasively monitored ICP and noninvasive B4C waveforms were recorded simultaneously from patients with acute brain injuries. Data were analyzed using specific cutoff values for the estimated P2/P1 ratio (ranging from 0.8 to 1.4) and TTP (at 0.3) to assess their diagnostic accuracy. Sensitivity and specificity for detecting IH (ICP > 20 mm Hg) were determined based on these metrics.

**Results:**

The estimated P2/P1 ratio demonstrated a sensitivity of 92% and specificity of 19% at a threshold of 0.8, indicating high sensitivity for ruling out IH. At a ratio of 1.4, the specificity improved to 90%, suggesting its effectiveness for assessing IH. For TTP, a threshold of 0.3 was identified as the optimal cutoff, offering a specificity of 92%.

**Conclusions:**

The B4C System provides a viable, noninvasive approach to assessing IH. The study underscores the clinical utility of the P2/P1 ratio and TTP in detecting and ruling out IH, offering a significant alternative to invasive ICP monitoring methods.

## Introduction

Intracranial pressure (ICP) monitoring is an important parameter in neurocritical care for managing conditions such as traumatic brain injury (TBI), hydrocephalus, and intracranial hemorrhage. Traditionally, ICP is measured using invasive techniques, which involve inserting catheters into the brain’s ventricular or parenchymal spaces. These methods, although accurate, come with significant risks, including infection, hemorrhage, and damage to brain tissue [1].

Recent advancements have focused on noninvasive methods for ICP monitoring, driven by the need to reduce the risks associated with invasive procedures. Intracranial compliance (ICC), the brain’s ability to accommodate volume changes without significant pressure increases, plays a crucial role in this context. ICC is an important parameter that reflects the brain’s capacity to buffer against volume changes, maintaining ICP stability and preventing brain injury [2]. Despite its significance, ICC has not yet been widely adopted in clinical practice because of challenges in its measurement and interpretation [3].

Studies have shown that ICC can complement ICP measurements by providing additional insights into the brain’s compensatory mechanisms. For instance, a high ICC indicates a greater capacity to accommodate volume changes, whereas a low ICC suggests a limited ability to buffer against increases in intracranial volume, leading to potential ICP spikes and brain injury [4]. Furthermore, understanding ICC dynamics can aid in predicting the progression of brain conditions, such as the risk of intracranial hypertension (IH), and tailoring individualized treatment plans [3].

The assessment of ICC can also be enhanced by analyzing specific features of the ICP waveform. Notably, the P2/P1 ratio and time-to-peak (TTP) are critical indicators of ICC and intracranial dynamics. The P2 wave (tidal wave) follows the P1 wave (percussion wave) in the ICP waveform, and an elevated P2/P1 ratio suggests reduced ICC, indicating a brain’s diminished ability to compensate for increases in intracranial volume. Additionally, TTP, or the duration from the start of the ICP wave to its highest peak, provides information about the intracranial system’s responsiveness to pressure changes. Shorter TTP intervals are typically associated with high ICC, whereas longer intervals indicate lower ICC [3, 5].

A recent survey of global experts in neurocritical care highlighted the significant role of ICP waveform assessment in the management of neurocritical care patients. The survey findings indicated that waveform features, such as the P2/P1 ratio and TTP, are critical for tailoring individualized treatment strategies and improving patient outcomes [6].

This study aims to evaluate the feasibility and clinical performance of a novel noninvasive device for intracranial dynamics monitoring capable of deriving such ICC parameters, referred to as the brain4care (B4C) System, against the established invasive ICP method and to assess its ability to detect IH in patients with acute brain injuries. The significance of this research lies in its potential to provide a safer, noninvasive alternative for ICP monitoring and the management of the risk of IH, ultimately improving patient outcomes and providing reliable alternatives for noninvasive brain monitoring.

## Methods

### Study Design and Population

This is a retrospective analysis of data from prospective observational studies conducted independently at four centers in Brazil, the São Paulo University’s Hospital das Clinicas, the Federal University of São Paulo, Hospital e Pronto Socorro Dr. João Lúcio Pereira Machado, and Hospital Estadual de Emergência e Trauma Senador Humberto Lucena; one center in Portugal, the University of Porto’s São João Hospital; and one center in the United States, Stanford University. Although these studies were independently conducted, they followed a shared data collection protocol, which included both invasive and noninvasive ICP monitoring. The study’s clinical trial protocol received approval from the local ethics committee in each center, and informed consent was obtained from the patients’ legally authorized representatives. This study was performed according to the Strengthening the Reporting of Observational Studies in Epidemiology standards (https://www.strobe-statement.org/checklists/). This study includes both previously reported data and novel, unpublished data to provide a more comprehensive evaluation of the B4C System’s performance in detecting IH.

Patients admitted at each center from January 1, 2015, to January 1, 2024, were eligible for inclusion in this study if they had experienced an acute brain injury (ABI) requiring invasive ICP monitoring within the first 5 days of their hospital admission. Patients who had undergone decompressive craniectomy or exhibited signs of brain death were excluded from the analysis. For cases involving TBI, the guidelines for the high risk of brain herniation established by the Brain Trauma Foundation [7] were followed. In the case of subarachnoid hemorrhage, management procedures were similar, although it is important to note that specific guidelines for ICP management in nontraumatic ABI cases are currently lacking [8]. It is worth mentioning that patient care was conducted independently of this study, and the data obtained using the B4C System’s sensor were not used for clinical decision-making or patient management.

## Neuromonitoring

ICP was monitored via an fiberoptic intraparenchymal transducer (Raumedic, Munchberg, Germany) or through an external cerebrospinal fluid shunt according to clinical indications. The B4C sensor (brain4care, São Carlos, São Paulo, Brazil), a wearable system cleared by the Food and Drug Administration (number K201989), was used to simultaneously register noninvasive ICP waveform. Previous studies described its principle of operation in details [9]. In summary, the B4C System involves placing a highly sensitive pin in contact with the skin over the skull that detects micrometric pulsatile cranial expansions originating from ICP variations each cardiac cycle. The waveforms obtained using the B4C System have been consistently correlated with invasive ICP in multiple clinical studies [10–14].

The B4C System was placed in the frontotemporal region on the same side as the ICP probe implantation. Simultaneous recordings were made of invasive arterial blood pressure (ABP), ICP, noninvasive pulsatile cranial expansion waveforms (B4C), electrocardiogram, temperature, and oxygen saturation. The data collection of these signals consisted of several short recording sessions, each lasting at least 10 min, electronically acquired and synchronized from patient bedside monitors using a custom data collection system (brain4care, São Carlos, São Paulo, Brazil) at a sampling frequency of 250 Hz. During these sessions, strict monitoring by the investigator in charge was maintained to prevent any displacement of the B4C sensor, which could potentially compromise the quality of the signal [15].

Prior to the analysis, the data set underwent preprocessing steps to ensure data quality and compatibility, filtering to reduce the signal-to-noise ratio (SNR), and artifacts removal. The power spectral density is derived from the downsampled and detrended signal, and it is used to estimate the SNR. The SNR is computed as the ratio of signal energy within the fundamental frequency and its first three harmonic frequencies, with a 0.2-Hz margin around each harmonic, relative to the total band spectrum spanning 0.1 to 25 Hz. Signals with an SNR exceeding the threshold of 0.35 are considered to meet quality standards and are subjected to the mean pulse assessment. For downsampled signals, standardizing sample rates is essential to ensure uniformity across all signals, ensuring a consistent processing pipeline filtration regardless of its acquisition sample rates.

## Collected Data Validation

Retrospective data postprocessing involved a rigorous curation process to enhance the quality of the processed signal, ensuring the reliability and accuracy of subsequent analyses. Initially, the analytical software within the cloud performed several tasks, including data parsing, detrending, signal validation, signal filtering, inversion verification, pulse identification, artifact removal, pulse alignment, pulse averaging, and pulse parameter calculation.

After this process, the P2/P1 ratio and TTP were extracted on a beat-by-beat basis and averaged into 60-s windows using B4C’s proprietary algorithm for ICP waveform analysis. This approach leverages the physiological correlation between ABP and ICP waveforms, as both are influenced by cardiac cycle. Key points in the ABP waveform, such as the systolic peak and dicrotic notch, correspond to critical events in the ICP waveform, enabling the estimation of P1 and P2. For instance, the systolic peak in ABP aligns closely with the timing of P1, whereas the distance between systole and the dicrotic notch is used to approximate the timing of P2. By normalizing these timings to the total pulse duration, the algorithm can estimate P1 and P2 amplitudes [16].

## Statistical Analysis

The sensitivity and specificity of various thresholds for the P2/P1 ratio and TTP were calculated to assess their effectiveness in predicting IH, defined as ICP exceeding 20 mm Hg. For the analysis, Python programming language was used (version 3.10.12, *scikit-learn* package).

## Results

**Fig. 1:**
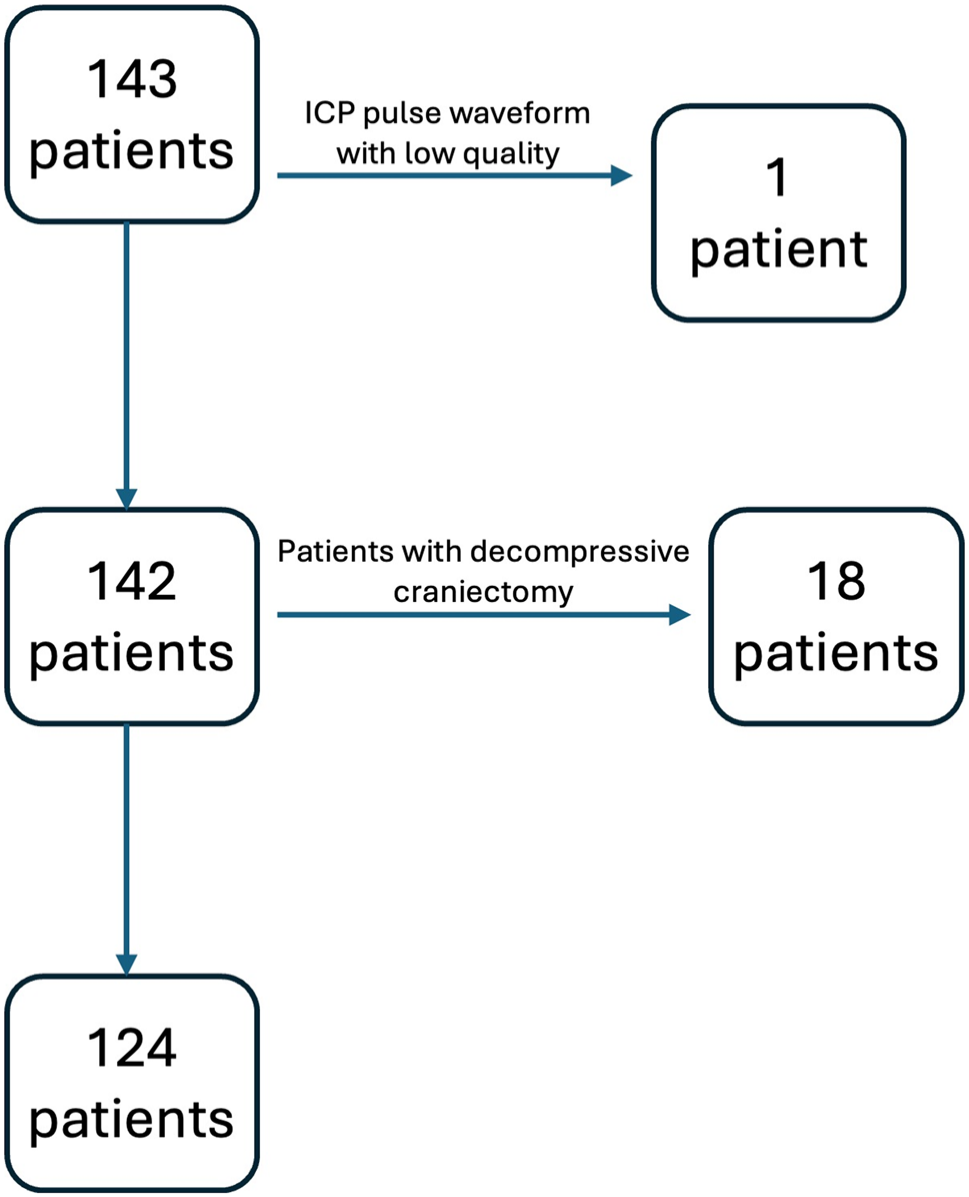
Flowchart of the data source and patient selection procedures. ICP intracranial pressure

A total of 143 patients were considered for assessment. One patient was excluded from the initial set because of the low-quality of pooled morphologies and short duration of monitoring, resulting in a revised pool of 142 patients. Subsequently, 18 additional patients presenting decompressive craniectomy were removed, resulting in 124 patients with 217 monitoring sessions whose data were segmented into 60-s windows, resulting in a total of 5,239 windows (Fig. 1). The averaged total monitoring time per patient was 42.3 ± 59.8 min. Overall, the entire population sample (*n* = 124) exhibited 9.7% of data with values above 20 mm Hg (Table 1). Table 1 represents the population sample demographics.

**Table 1:** Population demographic characteristics (*n* = 124)

	Overall
Sample (60-s windows)	5,239
Age, mean ± SD (years)	46.8 ± 18.0
ICP, mean ± SD (mm Hg)	11.6 ± 8.5
ABP, mean ± SD (mm Hg)	93.5 ± 17.2
*Sex, n*	
Male	82
Female	42
*Pathology, n*	
Traumatic brain injury	65
Subarachnoid hemorrhage	35
Intracerebral hemorrhage	14
Ischemic stroke	2
Mass	5
Subdural hematoma	1
Stroke	1
Other	1
*Site, n*	
São Paulo University’s Hospital das Clinicas	59
Federal University of São Paulo	6
Hospital e Pronto Socorro Dr. João Lúcio Pereira Machado	7
Hospital Estadual de Emergência e Trauma Senador Humberto Lucena	7
University of Porto, São João Hospital	11
Stanford University	34

## Performance of B4C System for Predicting and Ruling out IH

The B4C System was evaluated for its effectiveness in predicting and ruling out IH using two key parameters: the estimated P2/P1 ratio and normalized TTP. The performance metrics, including sensitivity and specificity, were analyzed at various cutoff values to determine the optimal thresholds for clinical application (Tables 2 and 3).

**Table 2:** P2/P1 ratio for sensitivity and specificity

P2/P1 cutoff	Sensitivity, %	Specificity, %	Interpretation
0.8	**92**	19	**High sensitivity for ruling out IH**
0.9	85	29	Moderate sensitivity and specificity
1.0	72	40	Balanced sensitivity and specificity
1.1	57	55	Moderate balance
1.2	36	68	Higher specificity, lower sensitivity
1.3	22	82	High specificity, low sensitivity
1.4	11	**90**	**High specificity for confirming IH**

IH, intracranial hypertension

**Table 3:** TTP thresholds for sensitivity and specificity

TTP cutoff	Sensitivity, %	Specificity, %	Interpretation
0.2	56	50	Balanced sensitivity and specificity
0.225	47	64	Higher specificity
0.25	31	74	Higher specificity, lower sensitivity
0.275	18	84	High specificity, low sensitivity
0.3	16	**92**	**Best cutoff for confirming IH**
0.325	9	96	Very high specificity, very low sensitivity
0.35	5	98	Very high specificity, very low sensitivity

IH, intracranial hypertension, TTP, time-to-peak

## Estimated P2/P1 Ratio

The estimated P2/P1 ratio demonstrated a range of sensitivity and specificity values depending on the cutoff employed. Notably, a cutoff of 0.8 provided the highest sensitivity (92%), making it an excellent parameter for ruling out IH. Conversely, a cutoff of 1.4 yielded the highest specificity (90%), indicating its utility in confirming IH (Table 2).

## Normalized TTP

Normalized TTP reflects the timing of waveform P1 and P2 peaks relative to the total pulse duration. Normalized TTP also showed varying sensitivity and specificity based on the cutoff values. A cutoff of 0.3 was identified as the best for predicting IH, with a specificity of 92%, making it highly effective for confirming the condition (Table 3).

## Discussion

The B4C System’s performance for IH prediction was evaluated in this study, focusing on two key parameters: the estimated P2/P1 ratio and normalized TTP. The findings feature the B4C System’s high sensitivity and specificity for predicting and ruling out IH, showcasing its potential as a viable noninvasive alternative to traditional invasive techniques. These results also align with previous reports in the literature, affirming the utility of the B4C System’s parameters to reflect ICP variations reliably under diverse clinical conditions [10–14].

## Comparison of the B4C System’s Performance in Previous Studies

The B4C System has been validated in multiple studies, demonstrating a strong correlation between its noninvasive parameters (P2/P1 ratio and TTP) and invasive ICP measurements. As summarized in Table 4, reported area under the curve (AUC) values range from 0.79 to 0.9, with sensitivity reaching 93% and a negative predictive value of 97%, supporting the B4C System’s ability to rule out IH [10, 12, 13, 17, 18]. While variations exist across patient populations and methodologies, these findings reinforce B4C’s role as a reliable noninvasive ICP waveform assessment tool.

**Table 4:** Comparative analysis of the performance of noninvasive intracranial pressure methods to detect intracranial hypertension

Study	Population	Noninvasive method	Sensitivity	Specificity	AUC	Key Findings
*Performance comparison of Brain4care System*						
Brasil et al. [12]	41 patients with ABI	B4C (P2/P1)	Not reported	Not reported	0.9	P2/P1 cutoff of 1.2 predicted IH
Brasil et al. [10]	72 patients with ABI	B4C (P2/P1)	60%	69%	0.88	AUC = 0.88 for predicting IH; AUC = 0.71 for early death
de Moraes et al. [13]	18 patients with stroke	B4C (P2/P1, TTP)	Not reported	Not reported	0.786 for P2/P1, 0.694 for TTP	Acceptable discriminatory power for noninvasive IH prediction
de Moraes et al. [17]	18 patients with ABI	B4C (P2/P1, TTP)	Not reported	Not reported	0.79 for P2/P1, 0.69 for TTP	Acceptable discriminatory power for noninvasive IH prediction
de Moraes et al. [18]	69 patients with ABI	B4C (P2/P1)	93%	60%	0.83	High negative predictive value (97%)
*Performance comparison of other noninvasive methods*						
Robba et al. [20]	Meta-analysis of 320 patients	ONSD	Not reported	Not reported	0.94	ONSD presents high accuracy for IH detection
Rasulo et al. [21]	262 patients with ABI	TCD	70%	72%	0.76	TCD-derived eICP IH threshold estimated at 20.5 mm Hg
Robba et al. [22]	195 patients with ABI	Pupillometry (NPi)	65%	70%	0.71	NPi < 4.1 associated with IH; moderate predictive power
*Performance comparison for the combination of noninvasive methods*						
Robba et al. [22]	195 patients with ABI	TCD-derived PI, TCD-derived eICP, ONSD, pupillometry	Not reported	Not reported	ONSD: 0.78; PI: 0.85; eICP: 0.86; NPi: 0.71	Combining ONSD and TCD-derived eICP improved accuracy (AUC = 0.91)
de Moraes et al. [17]	18 patients with ABI	TCD-derived PI + B4C	Not reported	Not reported	0.80	Multimodal combination of TCD-derived PI and B4C improved IH diagnostic accuracy
Brasil et al. [19]	98 patients with ABI	B4C + TCD	100%	3%	0.72	B4C and TCD-derived eICP showed AUC = 0.72 for predicting IH
Fernando et al. [23]	Meta-analysis of 5,123 patients	Various noninvasive ICP methods	Not reported	Not reported	ONSD: 0.94 (0.91–0.96), TCD-derived PI: 0.55–0.72	Pooling results across methods highlighted the highest predictive value for ONSD (AUC = 0.94)

ABI, acute brain injury; AUC, area under the curve; B4C, brain4care System; eICP, estimated intracranial pressure; ICP, intracranial pressure; IH, intracranial hypertension; NPi, Neurological Pupil Index; ONSD, optic nerve sheath diameter; PI, pulsatility index; P2/P1, ratio between the amplitudes of P2 and P1 peaks from the ICP waveform; TCD, transcranial Doppler ultrasonography; TTP, time-to-peak index

## Comparative Analysis of Sensitivity and Specificity for Different Noninvasive Methods

Other noninvasive methods, including optic nerve sheath diameter (ONSD), transcranial Doppler (TCD), and pupillometry, have been evaluated for IH detection, with their comparative performance detailed in Table 4. Meta-analyses report that ONSD has the highest predictive accuracy (AUC = 0.94) for IH detection [20], whereas TCD-derived estimated ICP shows moderate performance (AUC = 0.76) [21]. The Neurological Pupil Index has demonstrated moderate sensitivity and specificity (AUC = 0.71), but recent findings from the ORANGE study [15] challenge its clinical reliability because of variability across patient populations and its susceptibility to non-ICP-related factors.

## Combination of Noninvasive Methods for IH Detection

Multimodal approaches have been shown to improve diagnostic accuracy. As summarized in Table 4, combining ONSD and TCD-derived estimated ICP yielded the highest AUC (0.91), whereas integrating B4C and TCD achieved an AUC of 0.80–0.72, reinforcing the advantage of multimodal strategies for IH detection [17, 19, 22, 23].

## Clinical Interpretation of the B4C System’s Performance

To accurately interpret the B4C System’s findings related to screening for IH, it is essential to consider data from both patients undergoing critical care and healthy individuals, which can help establish normative thresholds. Recently, a study involving 188 healthy study participants across the age spectrum without significant comorbidities identified critical P2/P1 ratio thresholds that could serve as reference points in clinical protocols [24].

First, an estimated P2/P1 ratio of 0.8 or lower (Table 2) shows a sensitivity of 92%, making it a strong indicator of the absence of IH. This threshold may help avoid unnecessary clinical examinations (e.g., imaging), especially in clinically stable patients. Conversely, a P2/P1 ratio of 1.4 or higher (Table 2) has a specificity of 90%, an indicator of the presence of IH, strongly suggesting the need for further diagnostic evaluations, such as imaging, even in stable patients or those with inconclusive imaging results. These thresholds are consistent with observed normal values in the healthy population, in which most individuals had P2/P1 ratios below 1.2, except for middle-aged (45–64 years old) and older (> 65 years old) adult women, who showed higher P2/P1 ratios with medians of 1.43 (interquartile range: 1.35–1.50) and 1.42 (interquartile range: 1.34–1.50), respectively [24].

Regarding TTP, no threshold was found to have good sensitivity for ruling out IH. However, a TTP value of 0.3 emerged as a reliable reference point for predicting IH, with a sensitivity of 92%. This threshold is considered a strong indicator of the presence of IH, given that TTP values for all ages fall below 0.3 in healthy individuals [24].

**Fig. 2:**
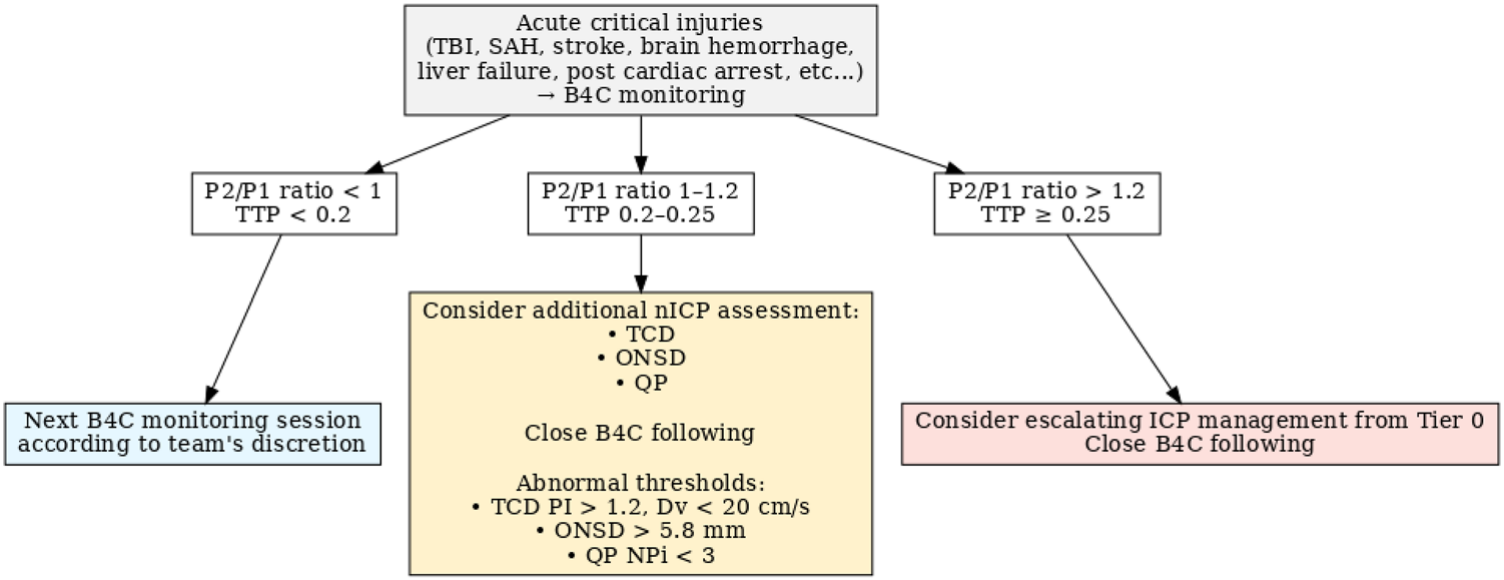
A proposed multimodal monitoring flowchart according to intracranial compliance parameters derived from the B4C System: P2/P1 ratio and nTTP. B4C brain4care, Dv diastolic cerebral blood velocity, ICP intracranial pressure, nICP noninvasive intracranial pressure, NPi Neurological Pupil Index, ONSD optic nerve sheath diameter, PI pulsatility index, QP quantitative pupillometry, SAH subarachnoid hemorrhage, TBI traumatic brain injury, TCD transcranial Doppler, TTP time-to-peak

The interpretation of P2/P1 values between 0.8 and 1.4 (Table 2) can be more nuanced. In this range, there is no clear threshold with sufficient sensitivity or specificity to definitively confirm or exclude IH; on the other hand, this range may be used for ICC dynamics trending and management. This variability can be attributed to individual differences in ICC, reflecting intrinsic physiological differences across sex and age groups. Therefore, it requires careful interpretation by trained clinicians, considering the patient’s overall clinical condition in a multimodal assessment approach (Fig. 2). In this context, recent findings from the study by Uysal et al. [25] in patients with ABI provided evidence to support the practical application of the B4C System as a noninvasive method for estimating ICC at the bedside. Their study indicated that noninvasive ICP waveform analyses offered a more accurate ICC estimate than TCD-derived methods for noninvasive ICP monitoring.

## Limitations

Patients with large skull defects, such as those resulting from craniectomies, may experience altered intracranial dynamics that could impact the accuracy of the B4C System [13]. Despite this, the value of ICP pulse morphology in these cases suggests that the B4C System could still offer meaningful insights [26]. A key limitation is its sensitivity to patient agitation, which can interfere with waveform readings, making it essential to minimize patient movement to optimize performance. Additionally, there is a risk of signal contamination from extracranial circulation pulsations if the sensor is incorrectly positioned near major extracranial arteries. However, proper sensor placement can prevent these artifacts. The B4C System includes built-in safeguards, such as an app algorithm that detects and alerts users to inadequate signals caused by improper placement or excessive artifacts, ensuring the collection of reliable data for clinical use.

The data set includes a limited number of observations with ICP values above 20 mm Hg, which may affect the statistical power for evaluating the B4C System’s performance at this and higher thresholds. Although the findings support the potential of the B4C System for IH detection, a larger sample of elevated ICP events would help further refine its accuracy across a broader range of clinical scenarios. Additionally, the absence of patient ventilation parameters data, such as partial pressure of CO_2_ (PaCO_2_), limits the ability to account for its potential influence on cerebral vessel dynamics and ICC. Because PaCO_2_ variations can impact ICP and waveform morphology, its inclusion in future studies may provide a more comprehensive understanding of the factors influencing B4C parameters and their interpretation in different clinical contexts.

## Conclusions

The B4C System shows excellent potential as a noninvasive tool for ruling out and detecting IH. Its high sensitivity and specificity for the P2/P1 ratio and TTP parameters provide a viable alternative to traditional invasive methods and other noninvasive technologies. The consistency of our findings with previous studies further strengthens the efficacy of the B4C System, making it a useful tool for noninvasive ICP monitoring and management of the risk of IH in various clinical conditions.

## Data Availability

The data that support the findings of this study are available from the corresponding author upon reasonable request.
